# Inflammatory cytokines and cardiac arrhythmias: from pathogenesis to potential therapies

**DOI:** 10.1097/MS9.0000000000003499

**Published:** 2025-06-16

**Authors:** Emmanuel Ifeanyi Obeagu

**Affiliations:** Department of Biomedical and Laboratory Science, Africa University, Mutare, Zimbabwe

**Keywords:** cardiac arrhythmias, inflammation, inflammatory cytokines, pathogenesis, therapeutic strategies

## Abstract

Cardiac arrhythmias, including atrial fibrillation and ventricular arrhythmias, are significant contributors to cardiovascular morbidity and mortality. Recent research has highlighted the critical role of inflammation in the pathogenesis of these arrhythmias, with inflammatory cytokines acting as key mediators. Cytokines such as interleukin-1, interleukin-6, tumor necrosis factor-alpha, and interleukin-17 are involved in promoting myocardial fibrosis, ion channel dysfunction, and autonomic dysregulation, which contribute to arrhythmic events. This review explores the relationship between inflammatory cytokines and cardiac arrhythmias, focusing on their molecular mechanisms, impact on heart tissue remodeling, and the role they play in arrhythmogenesis. Cytokine-induced inflammation leads to electrical and structural changes in the myocardium, which predispose the heart to arrhythmias. Elevated levels of pro-inflammatory cytokines can cause fibrosis, alter ion channel activity, and impair the normal conduction of electrical impulses. Additionally, cytokines enhance autonomic dysfunction, further increasing the risk of arrhythmia development. These findings underscore the significance of inflammation in the onset and progression of cardiac arrhythmias, particularly in conditions such as heart failure and atrial fibrillation, where persistent inflammation is prevalent.

## Introduction

Cardiac arrhythmias are abnormal heart rhythms that can significantly affect cardiovascular health, leading to morbidity, mortality, and impaired quality of life. These arrhythmias can arise from a variety of underlying causes, including structural heart disease, electrolyte imbalances, and ischemic heart conditions. However, growing evidence has increasingly pointed to the pivotal role of inflammation in the initiation, progression, and maintenance of cardiac arrhythmias. Inflammation in the heart can alter the normal electrical conduction system, promote fibrosis, and disturb ion channel function, all of which contribute to arrhythmic events. Central to this inflammatory process are cytokines, which are small proteins released by immune cells that regulate various immune and inflammatory responses^[1–5]^. The relationship between inflammatory cytokines and cardiac arrhythmias has garnered significant attention in recent years. Pro-inflammatory cytokines such as interleukins (interleukin-1 [IL-1], interleukin-6 [IL-6]), tumor necrosis factor-alpha (TNF-α), and interleukin-17 (IL-17) are particularly important in the context of arrhythmogenesis. These cytokines are involved in myocardial remodeling, including fibrosis, structural changes in the heart, and electrical disturbances that predispose the heart to arrhythmic episodes. In conditions like atrial fibrillation, heart failure, and ischemic heart disease, the levels of these cytokines are often elevated, promoting a chronic state of inflammation that is associated with poor arrhythmic outcomes[6].

**HIGHLIGHTS**

- Cytokine-induced arrhythmogenesis: Inflammatory cytokines like TNF-α, IL-1, and IL-6 contribute to cardiac arrhythmias by altering ion channel function, promoting fibrosis, and disrupting autonomic regulation.
- Molecular pathways: Nuclear factor-kappa B signaling, oxidative stress, and calcium dysregulation play key roles in cytokine-mediated electrical instability and arrhythmic events.
- Clinical associations: Elevated cytokine levels are linked to arrhythmias in conditions like myocarditis, heart failure, and post-myocardial infarction inflammation.
- Therapeutic approaches: Anti-inflammatory therapies, cytokine inhibitors, and immunomodulatory agents show promise in reducing inflammation-driven arrhythmias.
- Future perspectives: Precision medicine approaches targeting specific cytokines may lead to novel, personalized treatments for inflammation-induced cardiac arrhythmias.

Cytokines can directly influence myocardial cells, fibroblasts, and endothelial cells, leading to the remodeling of the heart’s structure. In particular, the induction of fibrosis by cytokines disrupts the extracellular matrix, creating scar tissue and altering the normal conduction of electrical impulses. This fibrosis is a hallmark of many arrhythmic conditions and is responsible for forming reentrant circuits that are crucial for arrhythmia development. Additionally, inflammatory cytokines can affect the ion channels that regulate the heart’s electrical activity, impairing normal depolarization and repolarization, and ultimately contributing to arrhythmias[7]. Autonomic dysfunction is another aspect of inflammation-mediated arrhythmogenesis. Cytokines can influence the autonomic nervous system, leading to an imbalance between sympathetic and parasympathetic activity. This autonomic dysregulation can further exacerbate arrhythmias, especially in conditions such as heart failure where both sympathetic overactivation and parasympathetic withdrawal are common. The interplay between inflammation, autonomic dysfunction, and arrhythmias underscores the complex mechanisms through which cytokines contribute to heart rhythm disturbances^[8–10]^. While much has been learned about the role of cytokines in the pathogenesis of cardiac arrhythmias, therapeutic approaches targeting these molecules are still in the early stages of development. There is growing interest in developing cytokine inhibitors and other anti-inflammatory therapies to prevent or treat arrhythmias. These therapies could help attenuate the inflammatory processes that lead to electrical remodeling and fibrosis, thereby reducing the risk of arrhythmic events. Current strategies include the use of biologics targeting specific cytokines, such as TNF-α inhibitors and IL-1 receptor blockers, with promising results in other inflammatory diseases[11]. The use of existing anti-inflammatory agents, such as statins, also shows potential in mitigating arrhythmogenesis[10]. Statins, while primarily used for their cholesterol-lowering effects, possess anti-inflammatory properties that may help reduce cytokine levels and prevent arrhythmias. Further research is needed to establish the efficacy of these therapies specifically in the context of arrhythmias and to determine the most appropriate patient populations for treatment^[12,13]^.

## Aim

This review aims to explore the complex relationship between inflammatory cytokines and cardiac arrhythmias, providing insight into the underlying mechanisms of cytokine-induced arrhythmogenesis and highlighting potential therapeutic strategies.

## Review methods

In this review, we aimed to synthesize the current understanding of the role of inflammatory cytokines in the pathogenesis of cardiac arrhythmias, explore the molecular mechanisms involved, and identify potential therapeutic strategies. A comprehensive approach was employed to gather relevant studies, focusing on both experimental and clinical data to provide an extensive analysis of the topic.

## Literature search and selection criteria

A systematic literature search was conducted using major scientific databases such as PubMed, Google Scholar, and Scopus, with an emphasis on articles published in the past decade. The search terms included “cytokines,” “cardiac arrhythmias,” “inflammation,” “TNF-α,” “IL-1,” “IL-6,” “arrhythmogenesis,” “treatment strategies,” and “anti-inflammatory therapies.” The inclusion criteria for studies were based on relevance to the topic, the quality of the study design, and the availability of robust data. We included peer-reviewed articles, clinical trials, meta-analyses, and experimental studies that investigated the relationship between cytokines and arrhythmias, as well as those exploring potential therapeutic interventions. Studies were excluded if they did not focus on inflammatory pathways or cardiac arrhythmias or if the data was limited to animal models without direct clinical implications.

## Limitations of the review

While this narrative review provides a comprehensive exploration of the involvement of inflammatory cytokines – particularly TNF-α, IL-6, IL-1β, IL-17, and TGF-β – in the pathogenesis of cardiac arrhythmias, several limitations must be acknowledged. First, as with many narrative reviews, there is an inherent selection bias in the literature included. Although a broad search strategy was employed, the absence of a systematic protocol means that some relevant studies might have been unintentionally excluded. This limits the reproducibility and completeness of the review process. Second, the article relies heavily on observational and preclinical studies, many of which demonstrate associations rather than causative relationships between cytokine activity and arrhythmogenic mechanisms. The translation of findings from animal models or in vitro experiments to human pathophysiology remains an ongoing challenge, and thus, conclusions drawn from such data should be interpreted cautiously. Additionally, the lack of quantitative synthesis (such as meta-analysis) limits the ability to provide objective statistical comparisons or pooled estimates regarding the prevalence, magnitude of risk, or therapeutic impact of targeting cytokines in arrhythmia management. This gap underscores the need for more rigorous, standardized reporting in future primary studies. Furthermore, while the review discusses emerging therapeutic strategies, many of these interventions – such as anti-TNF-α agents or TGF-β inhibitors – remain experimental or are only supported by limited clinical data. Therefore, the therapeutic recommendations presented should not be construed as clinical guidelines but rather as a call for further investigation. Finally, the heterogeneity of arrhythmia types (e.g. atrial fibrillation, ventricular tachycardia, and sudden cardiac death) and their multifactorial etiologies make it difficult to generalize the cytokine-mediated mechanisms across all cardiac arrhythmias. The review does not comprehensively differentiate the cytokine profiles or mechanistic pathways across these arrhythmia subtypes, which could be an important direction for future research.

## Inflammatory cytokines in cardiac arrhythmias

Inflammatory cytokines have emerged as critical players in the pathophysiology of cardiac arrhythmias. These small signaling molecules, produced primarily by immune cells such as macrophages, T lymphocytes, and endothelial cells, mediate inflammatory responses within the heart. As such, they have been implicated in the initiation, propagation, and maintenance of abnormal heart rhythms. Cytokines, including IL-1, IL-6, TNF-α, and IL-17, are key contributors to the inflammatory environment that alters the electrical and structural properties of the myocardium, fostering an arrhythmic substrate[14]. The role of inflammatory cytokines in cardiac arrhythmias is particularly evident in diseases such as atrial fibrillation, heart failure, and ischemic heart disease. Elevated levels of these cytokines in these conditions promote myocardial fibrosis, apoptosis, and alterations in ion channel function, which are critical for arrhythmogenesis. Cytokines, such as IL-1, IL-6, and TNF-α, are known to stimulate the activation of various signaling pathways, including the nuclear factor-kappa B (NF-κB) and mitogen-activated protein kinase pathways, both of which contribute to cardiac remodeling and arrhythmia development[15]. In particular, IL-1 is recognized as a potent mediator of inflammation in the heart. Its effects on the heart include the promotion of atrial fibrosis and the modification of ion channel expression, both of which contribute to the vulnerability of the myocardium to arrhythmias. Elevated IL-6 levels are also commonly found in patients with atrial fibrillation, and this cytokine is believed to influence electrical conduction in the heart by altering gap junctions and intercellular communication. Additionally, TNF-α has been shown to impact myocardial cells by inducing the release of reactive oxygen species (ROS), which in turn causes oxidative stress and ion channel dysfunction, further promoting arrhythmias[16]. Atrial fibrillation, one of the most common arrhythmias, is particularly associated with a persistent inflammatory state. The presence of elevated inflammatory cytokines in the atria has been linked to the progression from paroxysmal to persistent atrial fibrillation, as well as to the recurrence of arrhythmic episodes after ablation therapy. Similarly, in heart failure, where chronic inflammation is a hallmark, inflammatory cytokines contribute to the development of both atrial and ventricular arrhythmias. Studies have demonstrated that the suppression of inflammatory cytokine signaling in heart failure can reduce the incidence of arrhythmic events, thus providing evidence of the therapeutic potential of targeting these cytokines^[17–19]^. Moreover, cytokines have also been implicated in the modulation of autonomic nervous system activity, which in turn influences arrhythmia risk. Cytokines such as IL-6 and TNF-α affect the balance between sympathetic and parasympathetic tones, leading to heightened sympathetic activity and increased susceptibility to arrhythmic events. The inflammatory state in the myocardium alters the response to autonomic signaling, creating more excitable cardiac substrate^[20,21]^. Therapeutic strategies targeting inflammatory cytokines are a promising area of research for the treatment of cardiac arrhythmias. The use of cytokine inhibitors, such as IL-1 receptor antagonists, TNF-α blockers, and IL-6 inhibitors, has shown potential in other inflammatory diseases, and their application in arrhythmia management is under investigation. In addition, non-biologic agents, such as statins, which possess anti-inflammatory effects, have been suggested as potential adjunctive therapies for arrhythmia prevention, as they reduce cytokine levels and improve endothelial function (Table 1)^[22,23]^.

**Table 1:** Cytokines and their roles in cardiac arrhythmias

Cytokine	Mechanism of action	Evidence from studies
TNF-α	Downregulates connexin 43, disrupts gap junctions, prolongs repolarization	Elevated in AF and ventricular tachycardia patients
IL-6	Promotes myocardial fibrosis, prolongs QT interval	Associated with poor cardiac outcomes and arrhythmias in heart failure
IL-1β	Increases sympathetic tone, induces myocardial inflammation	Correlates with arrhythmia burden in systemic inflammation
IL-17	Enhances neutrophil infiltration, promotes electrical instability	Linked with autoimmune arrhythmogenic cardiomyopathy
TGF-β	Induces structural remodeling via fibroblast activation	Key mediator in fibrotic substrate for arrhythmia in mice models

## Molecular mechanisms of cytokine-induced arrhythmogenesis

Cytokine-induced arrhythmogenesis represents a complex interplay between immune signaling, myocardial structural remodeling, and electrophysiological alterations. The molecular mechanisms through which cytokines contribute to arrhythmias are multi-faceted and involve a variety of signaling pathways that influence both the electrical and structural properties of the heart. These mechanisms are especially evident in inflammatory heart diseases such as atrial fibrillation, heart failure, and ischemic heart disease, where elevated levels of pro-inflammatory cytokines like IL-1, IL-6, TNF-α, and IL-17 are commonly observed. Cytokines exert their effects by promoting myocardial fibrosis, altering ion channel function, inducing oxidative stress, and modulating the autonomic nervous system^[24–26]^. One of the most well-understood pathways involves the activation of NF-κB, a key transcription factor that regulates the expression of several pro-inflammatory cytokines, chemokines, and adhesion molecules. When activated by cytokines such as TNF-α and IL-1, NF-κB induces the expression of additional inflammatory mediators, which contribute to tissue inflammation and fibrosis. In the heart, prolonged NF-κB activation results in the accumulation of extracellular matrix components, leading to myocardial fibrosis. This fibrotic remodeling is central to arrhythmogenesis, as it disrupts normal electrical conduction and promotes the formation of reentrant circuits, which are crucial for the maintenance of arrhythmic events. The fibrotic tissue impairs electrical signal propagation, increasing the likelihood of ectopic beats and arrhythmic foci^[27,28]^.

In addition to fibrosis, oxidative stress plays a significant role in cytokine-induced arrhythmogenesis. Cytokines such as TNF-α and IL-6 trigger the release of ROS in myocardial cells. ROS can modify the function of ion channels, especially those involved in the action potential, such as sodium, potassium, and calcium channels. These changes can lead to altered action potential duration, increased excitability, and a more pro-arrhythmic state. Furthermore, ROS can contribute to the degradation of gap junction proteins, such as connexins, which are essential for the synchronization of electrical signals between cardiac cells. Disruption of gap junctions leads to electrical uncoupling and is a well-established contributor to arrhythmogenesis, particularly in atrial fibrillation and ventricular arrhythmias^[25,29,30]^.

Cytokines also modulate ion channel function, another key mechanism of arrhythmogenesis. Elevated levels of IL-1, TNF-α, and IL-6 can alter the expression and activity of ion channels, such as voltage-gated sodium (NaV) channels, potassium (K+) channels, and calcium (Ca2+) channels. Cytokine-induced changes in ion channel function can shorten or prolong the action potential, impair repolarization, and increase the likelihood of ectopic pacemaker activity. For example, TNF-α has been shown to reduce the expression of certain potassium channels, leading to prolonged action potentials and increased susceptibility to arrhythmias. Additionally, inflammatory cytokines can influence calcium handling within the myocardial cells, further enhancing the risk of arrhythmic events. These changes in ion channel function are thought to be particularly important in the context of atrial fibrillation, where electrical remodeling in the atrial myocardium is a hallmark feature^[31,32]^. Another mechanism by which cytokines contribute to arrhythmogenesis is through autonomic dysfunction. Inflammatory cytokines can influence the autonomic nervous system by promoting sympathetic overactivity and parasympathetic withdrawal, creating an imbalance that favors arrhythmias. IL-6 and TNF-α, for example, have been shown to increase sympathetic nervous system tone, which accelerates the heart rate and increases myocardial oxygen demand. Chronic inflammation can also impair the reflexive control of heart rate, further predisposing individuals to arrhythmic events. This autonomic dysregulation amplifies the electrophysiological disturbances induced by cytokines and contributes to the persistence of arrhythmias, particularly in heart failure and atrial fibrillation[33].

Electrophysiological remodeling is a critical feature of cytokine-induced arrhythmogenesis. Cytokines promote structural and electrical remodeling within the heart by inducing myocardial fibrosis, changing the expression of ion channels, and modulating the extracellular matrix. These changes lead to the development of a heterogeneous electrical substrate, where areas of the myocardium become more excitable and prone to ectopic impulses. This heterogeneity in electrical properties facilitates the formation of reentrant circuits and ectopic pacemaker activity, both of which are key mechanisms for arrhythmias. The remodeling of gap junctions, ion channels, and myocyte contractile proteins creates a permissive environment for the initiation and maintenance of both atrial and ventricular arrhythmias[25]. Finally, fibrosis and electrical remodeling are tightly linked in the progression of cytokine-induced arrhythmias. Myocardial fibrosis, induced by pro-inflammatory cytokines, not only disrupts the normal architecture of the heart but also impairs the electrical conduction system. Fibrosis alters the normal myocardial architecture, causing abnormal conduction pathways and increased anisotropy, which promote the development of arrhythmic foci. The fibrotic tissue itself becomes a source of electrical heterogeneity, enhancing the likelihood of arrhythmogenic events. This remodeling is particularly pronounced in the atria, where chronic inflammation is a key factor in the development of atrial fibrillation[8].

## Potential therapeutic strategies for cytokine-induced arrhythmias

The emerging role of inflammatory cytokines in the pathogenesis of cardiac arrhythmias has prompted the exploration of novel therapeutic strategies aimed at modulating the inflammatory response to reduce arrhythmia burden. Given that cytokines such as TNF-α, IL-1, and IL-6 contribute significantly to myocardial remodeling, ion channel dysfunction, oxidative stress, and autonomic dysregulation, targeting these pathways offers promising avenues for arrhythmia treatment. Several therapeutic strategies, including cytokine inhibitors, anti-inflammatory drugs, and modulators of specific signaling pathways, are being investigated for their potential to ameliorate inflammation-induced arrhythmias (Table 2)^[14,34]^.

**Table 2:** Potential therapeutic strategies targeting inflammatory cytokines

Therapeutic agent/strategy	Mechanism of action	Status/evidence
Anti-TNF-α agents (e.g. Infliximab)	Neutralizes TNF-α, reduces inflammation and electrical remodeling	Used in autoimmune diseases, promising results in myocarditis studies
IL-6R antagonists (e.g. Tocilizumab)	Blocks IL-6 receptor signaling, mitigates cytokine storm effects	Investigated in COVID-19-related myocarditis and arrhythmia
TGF-β inhibitors (e.g. Galunisertib)	Inhibits fibroblast-mediated remodeling and fibrosis	Preclinical studies in cardiac fibrosis models
Immunomodulators (e.g. corticosteroids)	Suppresses broad cytokine responses and myocardial inflammation	Clinically used, needs risk-benefit evaluation in arrhythmic patients
Targeted biologics (e.g. anti-IL-17 mAbs)	Interrupts IL-17 signaling, reduces immune-mediated tissue damage	Experimental, under evaluation for autoimmune cardiac syndromes

### Cytokine inhibition

One of the most direct approaches to reducing cytokine-induced arrhythmogenesis is the use of cytokine inhibitors. Biologic therapies targeting pro-inflammatory cytokines have shown efficacy in reducing inflammation and improving outcomes in several inflammatory diseases. For example, IL-1 receptor antagonists, such as anakinra, have been used to reduce the effects of IL-1, a potent inflammatory cytokine that contributes to fibrosis and electrical remodeling in the heart. In animal models and some clinical trials, IL-1 blockade has demonstrated a reduction in atrial and ventricular arrhythmias. Similarly, TNF-α inhibitors, including agents like infliximab and etanercept, have shown promise in reducing inflammation and improving cardiac function in heart failure patients, which in turn reduces arrhythmic events. These therapies not only reduce inflammatory cytokine levels but also target downstream pathways that contribute to myocardial fibrosis and electrical instability[35].

### Targeting oxidative stress

Oxidative stress is a key mediator of cytokine-induced arrhythmogenesis, particularly through its effects on ion channels and gap junctions. As inflammatory cytokines like TNF-α and IL-6 promote the generation of ROS, therapeutic strategies aimed at reducing oxidative stress hold potential in preventing arrhythmias. Antioxidants, such as N-acetylcysteine and Coenzyme Q10, have been shown to reduce ROS production and improve mitochondrial function. These agents may mitigate the adverse effects of oxidative stress on myocardial cells, including the preservation of ion channel function and the prevention of fibrosis. Clinical studies evaluating the role of antioxidants in the management of arrhythmias are ongoing, with the goal of identifying agents that can reduce the oxidative burden and thus restore normal cardiac electrical activity[36].

### Statins and other anti-inflammatory agents

Statins, commonly used for their cholesterol-lowering effects, also have significant anti-inflammatory properties. These drugs inhibit the production of pro-inflammatory cytokines, such as IL-6 and TNF-α, through their effects on the mevalonate pathway, which is involved in the synthesis of isoprenoids essential for the function of small GTPases. Statins have been shown to reduce the incidence of atrial fibrillation and improve outcomes in patients with heart failure by modulating the inflammatory response and stabilizing the endothelium. Additionally, non-steroidal anti-inflammatory drugs and corticosteroids have been explored for their ability to reduce systemic inflammation, though their long-term use is often limited by side effects. Nevertheless, statins remain one of the most promising pharmacological options for reducing cytokine-driven arrhythmias in patients with chronic inflammatory conditions^[37,38]^.

### Modulation of autonomic nervous system

Cytokine-induced autonomic dysregulation, particularly the imbalance between sympathetic and parasympathetic nervous system activity, contributes to arrhythmias. Anti-inflammatory therapies that address autonomic dysfunction could, therefore, be an important strategy. Beta-blockers, commonly used in the management of arrhythmias, can reduce sympathetic tone and improve heart rate control, thereby reducing the risk of arrhythmia. Furthermore, agents such as ivabradine, which specifically modulate the funny current channel and reduce heart rate without affecting contractility, may offer a targeted approach to balancing autonomic activity. Interventions that reduce sympathetic nervous system overactivity or enhance parasympathetic tone hold promise in mitigating the arrhythmogenic effects of inflammation[39].

### Gene therapy and RNA-based interventions

Gene therapy and RNA-based approaches represent cutting-edge strategies for targeting the molecular underpinnings of cytokine-induced arrhythmias. For example, small interfering RNAs or antisense oligonucleotides targeting pro-inflammatory cytokines or their receptors could be used to specifically downregulate the expression of key inflammatory mediators such as TNF-α, IL-1, or IL-6. Additionally, gene editing technologies, such as CRISPR/Cas9, may hold potential for correcting genetic predispositions that lead to heightened cytokine production in the heart. Though still in the experimental phase, these therapies could offer highly specific and long-lasting solutions for managing cytokine-driven arrhythmias by directly modulating the molecular sources of inflammation^[40,41]^.

### Electrophysiological modulation

In addition to pharmacological and biological therapies, electrophysiological approaches such as catheter ablation may be utilized to manage arrhythmias in patients with underlying inflammatory conditions. Catheter ablation involves the targeted destruction of myocardial tissue that is electrically unstable or forms part of an arrhythmic circuit. In patients with atrial fibrillation, for instance, ablation of the pulmonary veins has been shown to reduce arrhythmia recurrence, especially in those with elevated inflammatory cytokine levels. While ablation does not directly target cytokine signaling, it can reduce the burden of arrhythmias, which in turn may lead to a reduction in inflammation over time[41].

### Therapeutic vaccines

Recent advances in immunology have raised the possibility of using therapeutic vaccines to modulate the immune response in arrhythmia management. For instance, a vaccine targeting the IL-1β receptor could potentially prevent the downstream effects of IL-1 in the myocardium, reducing fibrosis and electrical remodeling. Therapeutic vaccines may also help modulate the autonomic nervous system by regulating cytokine-induced inflammation, providing a long-term solution for patients at risk of chronic arrhythmias^[42,43]^.

## Conclusion

Inflammatory cytokines play a pivotal role in the pathogenesis of cardiac arrhythmias, with substantial evidence linking elevated levels of pro-inflammatory cytokines – such as TNF-α, IL-6, and IL-1β – to the initiation and progression of arrhythmic events in patients with heart failure, atrial fibrillation (AF), and myocardial infarction. These cytokines contribute to myocardial fibrosis, ion channel dysfunction, and electrical instability, all of which are critical for the development of arrhythmias. Studies have demonstrated that elevated TNF-α levels correlate with increased risk of ventricular arrhythmias, while IL-6 and IL-1β promote atrial remodeling, which predisposes individuals to AF. Recent clinical evidence underscores the value of targeting these inflammatory pathways for arrhythmia prevention and management. Anti-TNF-α therapies, such as infliximab and etanercept, have shown promising results in reducing arrhythmic events, although further investigation is needed to determine their widespread clinical utility. Similarly, IL-6 inhibition with monoclonal antibodies like tocilizumab has shown potential in reducing AF incidence and improving outcomes in heart failure patients. Additionally, NLRP3 inflammasome inhibitors are emerging as promising therapeutic agents, with preclinical studies indicating their ability to reduce myocardial fibrosis and prevent arrhythmias. The objective value of these findings is evident in the potential to refine treatment strategies for patients at high risk of arrhythmias due to underlying inflammatory conditions. By targeting the cytokine-driven mechanisms of arrhythmogenesis, we can improve patient outcomes, reduce hospitalization rates, and potentially lower the mortality associated with cardiac arrhythmias. The integration of anti-inflammatory therapies with existing arrhythmic management could provide a more comprehensive approach, offering a novel avenue for therapeutic intervention.

## Data Availability

Not applicable as this is a narrative review.

## Research registration unique identifying number (UIN)

Not applicable as this is a narrative review.
